# A vision for estimation of the instantaneous reproductive number

**DOI:** 10.1016/j.epidem.2026.100885

**Published:** 2026-01-29

**Authors:** Chad W. Milando, George G. Vega Yon, Kaitlyn Johnson, Alessandra Urbinati, Guillaume St-Onge, Brennan Klein, Anne Cori, Laura F. White, with the Rt Collabathon participants

**Affiliations:** a Environmental Health, Boston University School of Public Health, Boston, MA, USA; b Center for Health Data Science, Boston University School of Public Health, Boston, MA, USA; c Affiliation Dept/Program/Center, University of Utah, Salt Lake City, UT, USA; d Department of Infectious Disease Epidemiology and Dynamics, London School of Hygiene and Tropical Medicine, London, United Kingdom; e Network Science Institute, Northeastern University, Boston, MA, USA; f Department of Physics, The Roux Institute at Northeastern University, Portland, ME, USA; g MRC Centre for Global Infectious Disease Analysis, School of Public Health, Imperial College London, London, UK; h Department of Biostatistics, Boston University School of Public Health, Boston, MA, USA; i Center on Emerging Infectious Diseases, Boston University, Boston, MA, USA

**Keywords:** Reproductive number, Surveillance, Software development

## Abstract

The reproductive number, $R_{t}$, is a popular metric used for monitoring infectious diseases. $R_{t}$ describes the expected number of infections that will be generated from a single infection at time t, which maps nicely to the likelihood that disease incidence will increase, decrease, or remain constant in the near future. Although this metric has existed for decades, it became more widely used during the COVID-19 pandemic and there was a subsequent proliferation of new estimation methods and software tools. This rapid development of methods and tools presents many opportunities and challenges for users, researchers, and decision makers. In recognition of this growth, we convened a three-day “collabathon” in September 2024 to bring together researchers and public health practitioners to identify challenges and areas for future development in $R_{t}$ estimation and to begin work in these areas. Here we provide a high-level summary of current methods and report on the findings from the collabathon, including a summary of current challenges and recommendations for future development, evaluation and interpretation of $R_{t}$.

## 1. Introduction

Infectious diseases continue to present a challenge to public health globally, as demonstrated by the COVID-19 pandemic, the emerging threat of novel strains of influenza, and other emerging and endemic diseases (Baker et al., 2022). Public health surveillance continues to be a key activity in managing and monitoring infectious disease threats and is increasingly aided by innovations in data availability, improved computing power, and increasing availability of methods to analyze data (Lipsitch et al., 2024; Brownstein et al., 2023; Jia et al., 2023; Donelle et al., 2023). Estimating current transmissibility provides information about the degree to which an epidemic is growing or in decline. A widely used measure of real-time transmissibility is the time-varying reproductive number, $R_{t}$, which estimates the average number of secondary cases generated by an infectious individual at a point in time (White et al., 2021; Gostic et al., 2020).

Accurate estimation and reporting of transmissibility can empower individuals to make decisions to protect their own health, and healthcare administrators and governments to make informed decisions about staffing needs and allocation of emergency resources (Lee et al., 2021; Dairi et al., 2021). Furthermore, during an emerging infectious disease outbreak, $R_{t}$ estimates provide valuable information about how close an epidemic is to the control threshold ($R_{t}=1$), which in turn can inform whether control is possible, and whether to tighten or loosen interventions (Lipsitch et al., 2003; Parag, 2021; Tsang et al., 2021). This quantity has been extensively described elsewhere (White et al., 2021; Gostic et al., 2020) and is often measured using reported case data at regular time intervals. If $R_{t}$ estimates are generated using a model that jointly infers over-dispersion (e.g., within a branching process model), they can also provide information about the role of superspreading, which in turn can guide behavioral interventions (Ho et al., 2023). These kinds of analyses have helped policymakers estimate how long it will take for an outbreak to reach extinction once it has been brought under control and interpret whether new case clusters are evidence of resurgence (White et al., 2009; Hollingsworth, 2009).

At the height of the COVID-19 pandemic in 2020, when $R_{t}$ was deployed to track the pandemic (Abbott et al., 2020; covidestim, 2025; rt.live, 2025), Gostic et al. (2020) described current challenges in implementing existing estimators relevant to reported case data (Cori et al., 2013; Bettencourt and Ribeiro, 2008; Wallinga and Teunis, 2004). They noted challenges and limitations in these existing methods, including the need to account for reporting delays in data and inconsistent application of biologically realistic data smoothing.

Since that study, there has been a proliferation of methods and software tools developed to estimate $R_{t}$ (Nash et al., 2022). Concurrent with this, there have been expanding data resources that could inform the estimation of $R_{t}$ methods, including wastewater, electronic healthcare records, and pathogen genetic sequencing data (Bonaldi et al., 2023; Fox et al., 2022; Huisman et al., 2022a; Lipsitch et al., 2024). Additionally, data are available at finer spatial scales, and the increased availability of high-performance computing permits the development of more complex estimation methods. In the United States, the CDC maintains real-time estimates of $R_{t}$ for COVID-19 and influenza (CDC, 2024). California maintains a similar dashboard (calCAT, 2025), and other states collaborate with CDC to generate estimates for internal review.

The rapid development of $R_{t}$ estimators has created challenges and opportunities for users and decision-makers who are interested in using $R_{t}$. This may be a positive development to the extent that it has improved ease of implementation, accuracy, and computational performance. One of the goals of the collabathon was to compare the existing methods and provide some guidance on the best use cases for each of the methods and more recently we have developed a resource to provide this information (Milando and White, 2026). Users may often be left to deploy the method that they are aware of and able to implement with ease. The latter is often a function of the documentation available for the method, the user’s familiarity with the coding language used to implement the method, and the extent to which they have seen others use the method in other settings. We will describe the challenges involved with the task of comparing $R_{t}$ estimation methods to evaluate their accuracy.

Here we report on work from a three-day workshop convened from September 24–26, 2024 dedicated to the evaluation and development of $R_{t}$ estimation tools and methods. This was sponsored by the Epistorm Center of Insight Net, a network of centers funded through the US CDC. The workshop brought together researchers, public health practitioners, public health decision-makers, and students to identify challenges and areas of future work on $R_{t}$ estimation and begin working on these challenges. Participants in the workshop were primarily current residents of the United States and United Kingdom, reflecting perspectives predominantly of the global north. During the first day, participants engaged in small group activities focused first on building familiarity with existing tools and identifying limitations and challenges. Subsequent work was aimed at addressing priority areas identified on the first day. In preparation for this workshop, we conducted a survey of practitioners and researchers to identify challenges and priority areas for development. Here, we report on key findings from both the survey and workshop. Survey results are summarized in the Supplementary materials.

In this paper, we will first provide a high-level summary of the current methods available for $R_{t}$ estimation. We will then describe the key challenges in the development, evaluation, use, and interpretation of $R_{t}$ estimates and estimators that were identified in the survey and workshop. Finally, we will present a road map for work in the near future on the development, evaluation, and interpretation of $R_{t}$.

## 2. Summary of current methods

We focus here on data-driven estimators of the instantaneous reproductive number, $R_{t}$. The reproductive number is a latent quantity not observable at a population level and therefore must be estimated from observed data. Estimators typically take reported case data to infer a time-varying estimate of $R_{t}$. These estimators commonly require an estimate of the generation interval, the distribution of times between infection for infector/infectee pairs. The serial interval, which is the distribution of times between symptom onset for the infector/infectee pairs, is often used as a proxy for the generation interval (Gostic et al., 2020). However, the serial interval is only an unbiased estimator of the generation interval if estimated using appropriate methods (Park et al., 2021).

The general approach to estimation is based on the assumption that there is a direct relationship between the number of newly infected individuals on day t, I(t), and the reproductive number. In the renewal equation (Fraser, 2007; Cori et al., 2013; Gostic et al., 2020), this is expressed as a convolution via the generation interval, ω as follows:

(1) $$I_{t}=R_{t}\sum_{i=1}^{t}\omega_{i}I_{t-i}$$

where $\omega_{i}$ is the probability of a generation interval of length i. When the number of new cases and serial interval are assumed known, there is nothing to infer, and the reproductive number is directly related to incidence. However, in practice, it is impossible to directly observe all incident cases with an appropriate time stamp as assumed by the renewal equation and we only observe discrete counts of incomplete and lagged case count data. The collabathon identified five main challenges: (1) incomplete case reporting, (2) delays in case reporting leading to right censoring, (3) volatility in reporting from day to day, creating variability in daily case counts (e.g. day of week effects), (4) aggregation of case data at a time scale incompatible with the generation interval, and (5) use of surrogates for infection data, such as symptom onset or reporting dates, that lag infection data. These limitations are the key motivations for available methods to estimate the reproductive number, with most methods focusing on addressing at least one of these limitations in case reporting data (Table 1). Methods to address case reporting delays are often referred to as nowcasting or right truncation adjustments and focus on filling in not-yet-observed cases to improve real-time estimates. Temporal smoothing approaches have been used to address the volatility of daily case report counts (Liu et al., 2024; Nash et al., 2023; Parag, 2021; Gressani et al., 2022; Abbott et al., 2020). Back-calculation techniques have been introduced to shift case report data backward in time to be more proximate to the date of infection so that $R_{t}$ estimates are appropriately aligned in time with true transmission dynamics (Abbott et al., 2020; Li and White, 2021). Fully generative modeling approaches handle this by modeling the forward process, from incident infections to expected observed cases, and fit directly to the observed cases.

Table 1 provides a summary of available software packages in the R coding language (Team, 2025) along with the challenges they aim to address. We focus on packages in R given their wide use among collabathon attendees, although we acknowledge there has been development in other languages recently, e.g., Python (Yang et al., 2021), Matlab (Chowell et al., 2024, 2016) and Julia (Abbott et al., 2026a; Song and Xiao, 2022).

We focus on methods that are based on time series of cases counts, but acknowledge that there is more emerging work to develop $R_{t}$ estimators that use alternative sources of data, such as wastewater data (Huisman et al., 2022b), hospital-based data (Fox et al., 2022), and mortality data (Na et al., 2020) to infer trends in cases and produce estimates of $R_{t}$. The methods we describe can reasonably be applied to hospital-based data but have also been used with wastewater data (Bonaldi et al., 2023; Fox et al., 2022; Huisman et al., 2022a).

## 3. Challenges

**Fig. 1.**
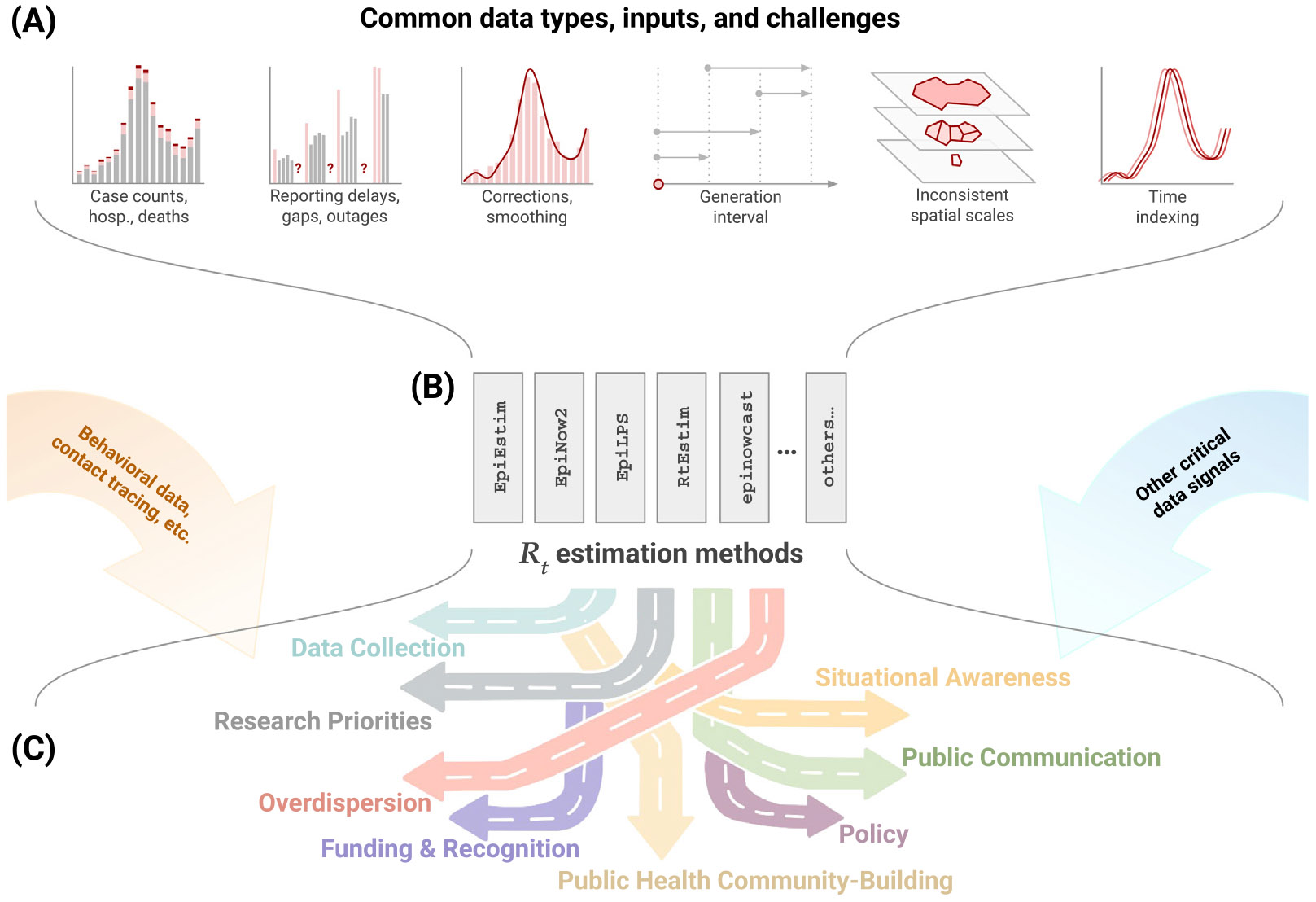
Illustration of the challenges and outcomes of $R_{t}$ estimation. Here, we illustrate a schematic pipeline of this process highlighting **(A)** several common challenges in data formatting and consistency, **(B)** examples of $R_{t}$ estimation tools (see Table 1), and **(C)** some of the many downstream tasks for which $R_{t}$ estimates are useful.

Here, we focus on the challenges of developing and implementing $R_{t}$ estimation methods and describe the subsequent obstacles practitioners may face in using $R_{t}$ to inform public health practice and decision-making in real time, as identified by workshop participants. Fig. 1 provides a visual overview.

### 3.1. Development

Some methods have been developed in response to a specific outbreak or challenge (e.g. Mukandavire et al. (2011), Ferreira et al. (2022), but it may be difficult to generalize these tools to other settings. This has implications in the two key dimensions of development that we highlight: (1) methods development and (2) software development and maintenance. An essential factor in the application of $R_{t}$ estimation in public health practice is the availability of well maintained tools and software. While advancements in $R_{t}$ estimation tools have democratized the availability high quality methods, additional maintenance, development, and documentation of these tools is needed to support accurate use and interpretation of the methods in public health practice. This section identifies some of these challenges.

#### Statistical challenges.

There are multiple statistical challenges to consider when estimating the latent transmission process, i.e. $R_{t}$, from an observable process (e.g. reported case counts, hospital admissions, etc.). There are typically multiple sources of uncertainty to consider in the estimation process, including uncertainty around the estimate of the generation interval and inconsistent, incomplete, aggregated and delayed case reporting (Nash et al., 2023, 2022). Appropriate quantification of these sources of uncertainty in final estimates is important to support valid interpretation of the estimates.

#### Insufficient academic incentives for professional software development practices.

Many innovative methods are published in peer-reviewed papers, and code is increasingly made available on GitHub or through R packages. Collabathon participants discussed challenges with inadequate documentation, lack of thorough testing, and unreliable dependencies. This is likely a reflection of the lack of incentives, crediting, and appreciation of software as a first-class scientific outcome (Pinto et al., 2018; Smith et al., 2018; Li et al., 2019).

#### Insufficient funding for maintenance and sustainability.

A common theme across scientific software is that after the initial development, there is a lack of support for continued maintenance of existing tools from most funding mechanisms (Carver et al., 2022). This means that existing software is sometimes maintained through pro bono time given by the original authors and the open-source community, which may lead to unreliable and inconsistent maintenance. Translating new methods into usable, well-tested, well-documented software may make the methods more accessible to public health practitioners.

#### Heterogeneous pool of users.

One of the significant challenges in public health methods and tool development is meeting the needs of a heterogeneous pool of users. Tools are often developed with a specific user in mind, but the end-user can vary widely in public health. The user base can be diverse including those interested in the technical aspects and looking for flexible implementations, as well as public health practitioners who need a more user-friendly, distilled product. Nonetheless, limited user feedback (Pinto et al., 2018) makes this issue more prominent.

### 3.2. Use and implementation

In this section, we focus on the user experience. The Collabathon brought together experts and trainees in infectious disease modeling, but still found that existing package documentation was not always sufficient to understand the modeling assumptions and parameter specifications of each package. This includes details on key assumptions and inputs, such as generation interval specification, time indexing, and smoothing which are not handled consistently between packages. We highlight some specific examples here.

#### Data inputs.

Most methods rely on the input of case counts and an estimate of the serial interval (Gostic et al., 2020; White et al., 2021; Wallinga and Lipsitch, 2007). However the formatting and preprocessing of these data can vary significantly between packages. Common points of difference include: how users are alerted to missing data, how dates and counts are formatted and named, how incomplete or right-truncated case data are incorporated into estimates, and how gaps in the time series are addressed.

#### Time indexing.

One challenge identified by workshop participants was inconsistency across packages in the definition of day 1 of an outbreak. In turn, inconsistent time indexing can make it unclear how the model outputs (estimated $R_{t}$) line up with the model inputs (cases). A related challenge is inconsistent indexing of delay parameters. For example, some packages (e.g., EpiEstim) require a leading 0 in the serial interval distribution, indicating a conservative assumption that symptom onset for an infectee cannot happen on the same day as symptom onset for an infector (Cori et al., 2013). At the time of this publication, some other packages do not have this restriction. Results from different packages sometimes lagged each other in ways that were challenging to understand using the available documentation.

#### Temporal smoothing.

Most packages employ some method to smooth data and/or estimates, based on the premise that data reporting is likely inaccurate at granular time scales. However, it can be challenging to determine the smoothing methods used by a package, and therefore challenging to interpret and compare their results. For instance, methods may assign the estimate of $R_{t}$ to the midpoint of a smoothing interval, but collabathon participants found it was not clear from the documentation on all methods if this is the case. This further contributes to the challenge of time indexing, with model inputs not clearly aligning with model outputs. Furthermore, packages differ in whether smoothness is set by the user (e.g. as a tuning parameter) or estimated jointly with $R_{t}$ (e.g. via the standard deviation of a random walk).

The implications of inconsistencies in the management of these issues in each software package and the lack of clear documentation may have ramifications for the user and interpretation of the results. The user typically must have significant technical knowledge and understand the method well, which may require direct communication with the developer to appropriately implement the method and interpret the results. This requirement is inconsistent with the goal of creating tools that can be implemented by users with varied backgrounds.

### 3.3. Evaluation

Inconsistency between $R_{t}$ estimates made when applying different tools to the same data and revision of real-time estimates as new data become available both make it difficult to interpret real-time estimates in practice. A recent study compared the performance of eight $R_{t}$ estimation approaches on COVID-19 data from Germany and showed that estimates made by different teams using different tools could differ substantially from one another, and that in some cases, the real-time estimates were revised significantly as additional data became available (Brockhaus et al., 2023). The study also importantly showed that differences between methods were largely attributable to the specification of the generation interval and smoothing window specification. In the experiments, the authors attempted to make assumptions and specifications as consistent as possible between methods to improve comparability, but still noted challenges in comparing the results of the different methods used in this exercise. This observation is consistent with results from a simulation study that showed different results between methods, particularly early in an outbreak when data is sparse (O’Driscoll et al., 2021). These observed discrepancies between methods could be seen as reinforcing the importance of using multiple methods simultaneously, i.e. ensembling, for decision-making (Wagenmakers et al., 2022) which is a practice in infectious disease forecasts endorsed and used by the US CDC (Loo et al., 2024; Cramer et al., 2022).

#### Comparability of $R_{t}$ estimates.

We have noted some of the challenges in comparing methods, including inconsistent time indexing and smoothing window definitions. These inconsistencies result in output $R_{t}$ estimates which may not be reliably comparable across methods at a given time point, t. Further, not all estimators estimate the same quantity (Gostic et al., 2020; White et al., 2021; Brockhaus et al., 2023). There are fundamentally different ways of conceptualizing the reproductive number, with the most commonly described approaches being the instantaneous reproductive number provided by the renewal Eq. (1), which is the most commonly used metric and our focus here (Gostic et al., 2020; White et al., 2021). The case reproductive number, which we do not focus on here, has a different definition and is not optimal for real-time estimation.

#### Data quality.

Studies that have attempted to compare $R_{t}$ estimates using real data provide useful insights on the relative performance of the methods, but lack the ability to identify the “best” method (Brockhaus et al., 2023). As noted previously, we only observe one realization of an inherently stochastic process, making it challenging to infer the underlying characteristics of the disease generating system and to disentangle this from data quality issues. It is well-recognized that case data are incomplete, delayed, and suffers from inconsistent reporting patterns (Herre et al., 2024; Long et al., 2022; Badker et al., 2021). However, it can be challenging to accurately model these phenomena. Many packages attempt to incorporate some or all of these features into estimation, relying on auxiliary data to inform reporting delays and incompleteness (Abbott et al., 2020). Even with these adjustments, the challenge of disentangling data quality issues from the accuracy of the estimates remains a challenge.

#### Evaluation metrics.

There are no agreed-upon metrics for evaluating $R_{t}$ estimates. Following (Brockhaus et al., 2023), ideally there are metrics that describe both the real-time accuracy of an estimator, and the comparative performance between methods. To date, most between- and within-method comparisons have been done visually (Gostic et al., 2020; O’Driscoll et al., 2021; Brockhaus et al., 2023), limiting the ability to catalog and succinctly summarize each method. For instance, these comparisons often visually show the performance of the methods across various simulation or real data examples, but do not systematically quantify performance. In forecasting, several metrics can be used to compare predictions to observed counts, including the weighted interval score (WIS), mean square error (MSE), and coverage probability (Reich et al., 2019; Lutz et al., 2019). Forecast scoring rules are not directly applicable to $R_{t}$ estimates because the true $R_{t}$ values are never directly observed in data. However, forecast-based evaluation could be applied to $R_{t}$ in other ways. For example, $R_{t}$ estimates could be used to generate one-generation ahead forecasts, and the forecasts could be scored. Alternatively, many $R_{t}$ methods generate estimates of latent cases, which can be scored directly against observed cases. We are not yet aware of any published application of forecast-based evaluation to $R_{t}$, but some $R_{t}$ estimation tools are designed to generate forecasts.

### 3.4. Decision-making

$R_{t}$ estimates can be a powerful tool to inform routine decisions made by individuals, healthcare administrators, and local or federal public health leaders. For all these stakeholders, estimates can be useful as a trend indicator that can help answer basic questions such as: “Has flu season started?”, “Has the current COVID-19 wave peaked?” and “Do I expect the situation to get better or worse in the next few weeks?”. Especially when used alongside information about current disease burden, trend indicators can help individuals make decisions about vaccination, social behavior, and masking. They also have potential to help healthcare administrators and decision makers plan staffing needs, vaccination campaigns, and supplies and resources. US CDC publishes weekly $R_{t}$ estimates for COVID-19 and influenza (CDC, 2024) to provide awareness of trends for these diseases. There remain challenges and opportunities to improve the ability to harness $R_{t}$ estimates for decision-making, as we describe in this section.

#### Discretizing $R_{t}$.

In public health practice, categorical outcomes are considered easier to interpret and more visually accessible to the public than continuous outcomes, so CDC reports trends by discretizing estimated posterior probabilities of epidemic growth into categories (CDC, 2024). Opportunities remain to further formalize methods to this end in a unified estimation framework with decision support being the ultimate outcome.

#### Reporting delays.

Effective use of $R_{t}$ estimates for decision support depends on their timeliness and accuracy. Reporting lags, latency, and outages in data reporting systems are currently some of the main obstacles to producing timely, accurate estimates. If reporting delays are routine and predictable, models that include the case observation process, and incorporate right truncation adjustments or nowcasting methods to adjust for incomplete reporting on recent dates, can provide timely $R_{t}$ estimates that are leading indicators relative to trend estimates based on unadjusted data (Richard, 2024).

#### Reporting outages.

Not all reporting delays are routine or predictable. For example, a software update, a cyber attack, or a fire at a data center could all cause incidental outages in surveillance data reporting. These incidental outages are particularly problematic if they persist for longer than a few days, and if they affect many jurisdictions or a large geographic area. Currently, CDC works with surveillance system partners to monitor total reported visit volumes and identify incidental reporting outages when generating $R_{t}$ estimates each week. If there is evidence that reported values are incomplete due to an incidental outage, $R_{t}$ estimates can be withheld for the affected jurisdiction until the data are corrected, or anomalous data points can be dropped and the $R_{t}$ estimation model can infer the missing values (CDC, 2024).

Methods for dealing with incidental reporting outages could be improved in two key ways. First, more automated model-based approaches would be able to accurately infer latent reports from incomplete values, reducing withheld estimates, and reliance on extrinsic monitoring or time-consuming manual data review. Second, adoption of methods that can see and learn from more than one data signal may be robust to incidental outages; if one signal becomes inaccurate for a period of time, the model may be able to draw accurate information from other signals.

#### Signal fusion.

Signal fusion, an approach where a single model is fit to multiple data sources (e.g., wastewater, emergency department visits, and hospitalizations), is a promising approach to improve decision-making. Wastewater-based forecasts, which combine wastewater data with more traditional epidemiological signals such as hospitalization counts, can be robust to data reporting anomalies, but can also return inaccurate results if the two signals disagree for a prolonged period of time (Hill et al., 2025, 2023).

#### Hierarchical models.

Another promising approach involves joint estimation of $R_{t}$ across many locations using a single data source. For example, if $R_{t}$ values for all locations were estimated jointly in a hierarchical model, then information from locations with complete reporting may help the model infer accurate values in locations where data are missing or incomplete. This partially pooled hierarchical approach has been used with some success by the UK Health Security Agency (UKHSA) (Ward et al., 2021). Additionally, finer spatial scale estimates incorporating mobility data and enabling more targeted interventions have been developed for EpiEstim (Zhou et al., 2022), but have not been widely used and tested. The limited availability of mobility data also hampers their implementation.

## 4. Roadmap

Discussions at the collabathon demonstrated that the community interested in the technical and practical aspects of estimating the epidemic reproduction number span both academia and public health agencies. Here, we propose a road map of our vision of the short- to medium-term priorities for $R_{t}$ estimation going forward.

### 4.1. Bringing together the community

Methods for $R_{t}$ estimation have predominantly been developed in the midst of an ongoing outbreak, most prominently, during the height of the COVID-19 pandemic (Alvarez et al., 2021; Gressani et al., 2022; Abbott et al., 2020; Champredon et al., 2024; Scire et al., 2023; Liu et al., 2024). Collabathon participants noted that the result has been a proliferation of methods that are challenging to compare and lack consistency in documentation and implementation. Some degree of coordination between developers to establish standards and exchange knowledge and experience could enable the production of methods and tools that are more suitable for a wide range of end users and more variable settings. We consider it important that this community encompass both researchers developing methods, software developers, and end users, including public health officials. Our experience in the collabathon was that having this breadth of individuals in the same space working collaboratively grounded discussions around future development in the practicalities of public health practice while communicating important problems that public health officials face.

We started building a community of individuals interested in $R_{t}$ estimation at the collabathon and hope to raise more awareness and participation in this nascent community through this written piece.

### 4.2. Establishing a suite of approaches and/or metrics to evaluate $R_{t}$

As we have described, there are no standard metrics for evaluating the performance of $R_{t}$, nor is there consensus on the correct way to evaluate $R_{t}$. We have described multiple aspects that are important to consider in this exercise, including (1) real-time estimation performance, (2) between-method consistency, and (3) usability metrics (computational speed, ease of use, documentation, software maintenance, etc.). The best metrics to evaluate $R_{t}$ may depend on the intended use of $R_{t}$.

A group at the collabathon began to explore and develop the idea of using forecast-based metrics to evaluate $R_{t}$, but further work is needed.

### 4.3. Recognition for scientific software development

Recognizing scientific software as a first-class scientific contribution may help improve the usability and accessibility of tools for $R_{t}$ estimation, for example through motivating better documentation and user support. Collabathon participants from academia expressed interest in this kind of work, but noted that scientific software development is not typically recognized or incorporated into academic career progression systems (Puebla et al., 2024; Iyamu et al., 2022).

### 4.4. Ensuring usability and sustainability of software

Currently, there are no community-defined standards in software development for $R_{t}$ estimation and developers at the event indicated they have found limited support for the maintenance of existing software. As described, documentation is often insufficient, and inputs and outputs are inconsistent between packages, hampering valid comparisons between methods and limiting the user base. We propose the development of a checklist of potential features for $R_{t}$ estimation software that developers would be encouraged to reference and indicate which features their software contains. This checklist would include both methodological options (*e.g.*, accounting for reporting delays) and implementation features, including questions about installing the software, documentation, computational efficiency, dependencies, etc. Along with this checklist, we propose developing a standard set of output formats that developers would be encouraged to adopt. For example, to facilitate comparisons of output across packages, the standard outputs may include nowcasting and forecasting functionality, as well as methods for handling missing data.

Participants in the collabathon also identified the need for comparison methods between tools and improved usability. First, participants indicated that tools should provide a sampler to easily generate a sample of $R_{t}$ estimates. Second, including an epidemic simulator or projection approach may be desirable to enable evaluation with forecasting metrics. In addition, a large pool of data from real outbreaks in different locations with different public health interventions implemented at varying stages of the outbreak could be a useful resource in comparing performance and identifying meaningful differences in estimators in real world settings. The RECON project is taking initial steps to collate this type of data (Consortium, 2025). Finally, users may benefit from improved documentation.

### 4.5. Formally comparing existing methods and tools for $R_{t}$ estimation

As described above, evaluation of $R_{t}$ estimates is hampered by a lack of agreed-upon metrics and inadequate documentation in existing methods to enable comparisons.

#### Current progress.

As outlined in Table 1 we have started a high-level summary of existing methods. Further development of a process to harmonize software outputs may help achieve a more formal comparison, which we propose as a key milestone for our community. However, much work remains to more formally compare existing methods with an eye toward their ultimate intended use in practice.

### 4.6. Maximizing translational impact

$R_{t}$ gained traction as a popular and helpful metric to monitor the COVID-19 pandemic across the globe. During the pandemic response, active partnerships between modelers, software developers, and public health decision makers helped identify pitfalls in the existing tools, and this fueled rapid innovation to address those gaps. Maintaining and strengthening such partnerships may help academic methods and software development be more responsive to applied needs and decision making. Software documentation and maintenance are important facets of $R_{t}$ implementation, which do not always fit well into standard academic workflows.

### 4.7. Long-term vision

We have outlined a road map to initiate a coherent community-driven effort to help $R_{t}$ estimation methods and tools better serve public health needs in future epidemics and ongoing monitoring of infectious diseases. Improving pandemic responses requires that resources, including human resources and tools necessary for responses, are sustained. We can perhaps learn from other communities, such as that of epidemic forecasting, which, driven by annual flu-forecasting efforts, has been very active and collaborative over multiple years and leveraged this experience to stand up a COVID-19 forecasting hub in 2020. There are also potential opportunities for stronger collaboration between forecasters and the nascent $R_{t}$ estimation community to enable information sharing since $R_{t}$ estimates are used in some forecasting methods, and forecasting methods can also inform $R_{t}$ estimates.

## 5. Conclusion

We have described key takeaways from discussions during a collabathon convened in September 2024 with experts on $R_{t}$ estimation, including practitioners and academic researchers. We emphasize the need for a sustained community to guide the future development of $R_{t}$ estimation methods and software, establish standards for the implementation and evaluation of software and methods, and coordinate with other related efforts for infectious disease monitoring. We also recognize the need to support multidisciplinary partnerships that focus on creating and maintaining tools and software that impact public health practice and enable data-informed decision making.

## Supplementary Material

1

## Figures and Tables

**Table 1:** Summary of available packages in R for $R_{t}$ estimation. Packages included that have updates after June 1, 2023.

Package	Description	GitHub URL
EpiEstim (Cori et al., 2013)	Assumes a Gamma prior distribution for $R_{t}$, and a Poisson distribution for new cases, which permits an analytical solution for the posterior distribution of $R_{t}$. Uses fixed-size sliding windows to smooth $R_{t}$ over time.	mrc-ide/EpiEstim
EpiInvert (Alvarez et al., 2021)	An augmentation of the EpiEstim approach, by incorporating the case reproduction number formulation of $R_{t}$ to improve posterior accuracy.	lalvarezmat/EpiInvert
EpiLPS (Gressani et al., 2022)	Assumes cases follow a Negative Binomial distribution. Uses Bayesian P-Splines to smooth the case time-series, and then B-splines to estimate $R_{t}$ using the renewal equation.	oswaldogressani/EpiLPS
EpiNow2 (Abbott et al., 2020)	A fully generative Bayesian model that models infections and various delay distributions. Many options for $R_{t}$ generation, including Gaussian Process models and random walk models.	epiforecasts/EpiNow2
epinowcast (Abbott et al., 2026b)	A fully generative Bayesian model that models infections and various delay distributions. The base model uses a random walk model to constrain $R_{t}$ in time. Aims to address some of the limitations of EpiNow2, e.g., by adding more capacity for modularization	epinowcast/epinowcast
ern (Champredon et al., 2024)	A wrapper for EpiEstim that contains various delay distributions. Similar to esimateR, cases are first smoothed using LOESS, then deconvolved backwards in time to produce an infections time-series.	phac-nml-phrsd/ern
estimateR (Scire et al., 2023)	A wrapper for EpiEstim that contains various delay distributions. Similar to ern, cases are first smoothed using LOESS, then deconvolved backwards in time to produce an infections time-series.	covid-19-Re/estimateR
RtEstim (Liu et al., 2024)	Uses the renewal equation and Poisson trend filtering on the divided difference of adjacent $R_{t}$ values. This produces discrete splines for $R_{t}$.	dajmcdon/rtestim
tsgc (Ashby et al., 2024)	Non-renewal equation based estimate of $R_{t}$, made by smoothing cases using a Kalman filter applied to a dynamic Gompertz model	Craig-PT/tsgc

## Data Availability

No data was used for the research described in the article.

## Appendix A. Supplementary data

Supplementary material related to this article can be found online at https://doi.org/10.1016/j.epidem.2026.100885.
