# Supplementary material for: A vision for estimation of the instantaneous reproductive number
Source: Epidemics. Author manuscript; Available in PMC 2026 Apr 14. (PMC13077567; doi:10.1016/j.epidem.2026.100885)
Supplement: 1 [file NIHMS2157303-supplement-1.pdf]

hospitalizations superior to cases and test positivity: A prediction study. Infectious Disease Modelling. 2023;8(4):1138–1150. doi:10.1016/j.idm.2023.10.004.

63. Ward T, Glaser A, Johnsen A, Xu F, Hall I, Pellis L. Growth, reproduction numbers and factors affecting the spread of SARSCoV-2 novel variants of concern in the UK from October 2020 to July 2021: a modelling analysis. BMJ Open. 2021;11:e056636. doi:10.1136/ bmjopen-2021-056636.

64. Zhou Z, Kolaczyk ED, Thompson RN, White LF. Estimation of heterogeneous instantaneous reproduction numbers with application to characterize SARS-CoV-2 transmission in Massachusetts counties. PLoS computational biology. 2022;18(9):e1010434. doi:10.1371/journal.pcbi.1010434.

65. Alvarez L, Colom M, Morel JD, Morel JM. Computing the daily reproduction number of COVID-19 by inverting the renewal equation using a variational technique. Proceedings of the National Academy of Sciences. 2021;118(50):e2105112118. doi:10.1073/pnas.2105112118.

66. Champredon D, Papst I, Yusuf W. ern: An R package to estimate the effective reproduction number using clinical and wastewater surveillance data. PLOS ONE. 2024;19(6):e0305550. doi:10.1371/journal.pone.0305550.

67. Scire J, Huisman JS, Grosu A, Angst DC, Lison A, Li J, et al. estimateR: an R package to estimate and monitor the effective reproductive number. BMC Bioinformatics. 2023;24(1):310. doi:10.1186/s12859-023-05428-4.

68. Puebla I, Ascoli GA, Blume J, Chodacki J, Finnell J, Kennedy DN, et al. Ten simple rules for recognizing data and software contributions in hiring, promotion, and tenure. PLOS Computational Biology. 2024;20(8):e1012296. doi:10.1371/journal.pcbi.1012296.

69. Iyamu I, Gómez-Ramírez O, Xu AX, Chang HJ, Watt S, Mckee G, et al. Challenges in the development of digital public health interventions and mapped solutions: Findings from a scoping review. Digital Health. 2022;8:20552076221102255. doi:10.1177/20552076221102255.

70. Consortium RRE. R Epidemics Consortium;. Available from: `http://reconhub.github.io/`.

Supplemental Information

1 Planning and executing a collabathon

The 2024 Epistorm Collabathon was structured as an intensive, interdisciplinary research sprint aimed at improving R_t estimation methods while providing a scalable model for structured, time-bound scientific collaboration. The event was designed around three distinct but interdependent phases: a pre-collabathon preparation period that established a foundation of shared knowledge and research priorities, a semi-structured, in-person, collaborative phase that balanced formal discussion with hands-on problem-solving, and a post-collabathon sustainability strategy to ensure that the projects initiated during the event would continue to develop. This design was guided by the principle that short-term, high-intensity collaborations can accelerate methodological advances while fostering long-term research communities. We believe that this structured approach may provide a framework that can be adapted for future collabathons across different fields, particularly those that require computational tool

development, methodological benchmarking, and interdisciplinary expertise; below, we aim to share a blueprint for running events like this.

1.1 Pre-Collabathon Preparation

The effectiveness of a collabathon is largely determined by the preparation that occurs beforehand. For the 2024 Epistorm Collabathon, organizers prioritized a structured approach to pre-event planning to ensure that participants arrived with a common foundation of knowledge and that the event itself could focus on implementation rather than introductory discussions. The first step in this process was targeted outreach to researchers, developers, and public health practitioners actively working on R_t estimation and real-time epidemic modeling. Rather than imposing a strict research agenda to begin with, organizers sought to shape the event around the most pressing challenges identified by prospective participants. To this end, a survey was distributed in the months leading up to the collabathon, soliciting input on key technical barriers, unmet needs in the field, and specific limitations of existing R_t estimation tools. The BUMC Institutional Review Board determined that this was not human subjects research.

The responses to our survey revealed several recurring themes, including the absence of standardized benchmarking frameworks, inconsistencies in input formats across different estimation software, computational inefficiencies in real-time applications, and the difficulty of adapting R_t estimation tools for practical decision-making in public health. These responses directly informed the structure of the collabathon, ensuring that discussions and working groups would be oriented toward well-defined, high-priority research problems.

To further facilitate productive engagement, organizers curated a set of preparatory materials designed to provide participants with a common technical foundation. These materials included a collection of relevant research papers, an overview of widely used R_t estimation methodologies, and a reference guide detailing the statistical assumptions underlying different approaches. In addition to these static resources, organizers developed an interactive RShiny-based dashboard that allowed users to manipulate key model parameters and observe the effects of different assumptions on R_t estimates. This tool was shared with participants in advance of the event and was later used as an anchoring point for discussions during the collabathon itself. The goal of this preparatory phase was not only to ensure a baseline level of technical fluency among participants but also to encourage pre-collabathon engagement with key methodological questions.

Beyond intellectual preparation, logistical considerations were also prioritized to maximize the efficiency of in-person collaboration. Participants were provided with access to shared coding environments, data repositories, and communication channels to enable real-time collaboration.

1.2 Collaborative Execution During the Collabathon

The in-person phase of the collabathon was designed to maximize the efficiency of interdisciplinary collaboration while allowing for both structured guidance and exploratory research. The event opened with a framing session led by Prof. Laura White and Dr. Chad Milando, in which they outlined the primary objectives of the collabathon, identified key gaps in R_t estimation methodologies, and introduced the core computational challenges to be addressed. This was followed by a live demonstration of the RShiny dashboard, which allowed participants to visualize how

different modeling assumptions, data constraints, and statistical choices influenced R_t estimates. This interactive session served to ensure that all participants had a common technical reference point before transitioning into more specialized discussions.

Following this introductory phase, participants self-selected into working groups based on their expertise and research interests. Unlike traditional workshops where teams may be pre-assigned, the self-organization model ensured that participants engaged with problems they found most compelling while maintaining a balance of technical expertise within each group. To prevent early-stage stagnation, facilitators played an active role in guiding discussions, ensuring that teams quickly moved from conceptual framing to well-defined research objectives.

Four major working groups emerged during the collabathon, each focusing on a distinct but interrelated challenge. The first group addressed evaluation and diagnostics, developing systematic approaches for assessing R_t estimation methods, with a particular focus on forecast-based evaluation and comparisons across different spatial and temporal scales. The second group worked on the development of `SummRt`, a software tool designed to harmonize the outputs of multiple R_t estimation methods, allowing for structured comparisons and benchmarking within a unified computational framework. The third group explored the feasibility of implementing core R_t estimation routines in Julia, with the goal of improving computational efficiency and numerical stability for real-time applications. The fourth group tackled the issue of missing data, developing an initial framework for inferring local R_t estimates from broader regional trends using hierarchical modeling techniques.

The second day of the collabathon was dedicated to focused research and development within these working groups. Teams refined their methodological approaches, tested preliminary solutions, and documented their progress in shared repositories to ensure continuity beyond the event. Periodic check-ins with facilitators provided opportunities for teams to troubleshoot technical challenges, refine their research plans, and integrate feedback from other participants. The combination of structured guidance and open-ended collaboration allowed for both depth and flexibility in problem-solving.

The final day of the collabathon was dedicated to synthesizing results and planning for long-term sustainability. Each working group presented its findings, detailing the technical progress made and outlining the next steps required to bring their projects to completion. This phase of the event was particularly important in ensuring that the work conducted during the collabathon would have a lasting impact rather than remaining a one-time research sprint. Facilitators led discussions on strategies for maintaining engagement beyond the event, emphasizing the importance of documentation, structured follow-up, and clearly defined research deliverables.

1.3 Post-Collabathon Sustainability and Long-Term Impact

A common limitation of short-term research sprints is that promising ideas often fail to gain traction once the event concludes. To mitigate this, the 2024 Epistorm Collabathon was designed with a structured post-event engagement strategy to ensure that projects initiated during the event would continue to develop.

The first priority was ensuring that all research outputs were properly documented and publicly available. Each working group maintained shared repositories throughout the event, ensuring that code, datasets, and methodological notes could be accessed and expanded upon. The `SummRt` team, for example, established a development roadmap for transforming their prototype into a fully functional benchmarking tool, while the

evaluation and diagnostics group proposed a formal framework for comparing different R_t estimation methods.

The second priority was sustaining community engagement. A dedicated Slack workspace was created to facilitate ongoing discussions, allowing participants to share updates, request feedback, and coordinate follow-up work. A series of virtual check-ins was scheduled in the months following the event, providing structured opportunities for teams to report on progress and troubleshoot challenges.

The third priority was securing institutional and financial support for long-term sustainability. Discussions during the collabathon highlighted the broader issue of limited funding for software maintenance in epidemiology, despite the critical role that computational tools play in public health decision-making. Participants explored potential funding avenues, including research grants, partnerships with public health agencies, and integration into larger forecasting initiatives.

By documenting the structure, process, and outcomes of the 2024 Epistorm Collabathon, we hope to provide a scalable framework for research groups interested in organizing similar collaborative sprints. The event demonstrated that, with careful planning and strategic facilitation, short-term, high-intensity collaborations can yield meaningful scientific advancements while fostering an engaged and sustainable research community. The principles that contributed to the success of this collabathon—problem-driven engagement, structured but flexible collaboration, and an emphasis on long-term sustainability—can be adapted across disciplines where computational research intersects with practical decision-making needs.

2 Pre-collabathon Survey

In July 2024, we developed and sent a survey to gain preliminary understanding of what R_t tools are being used and how they are being used. We were also interested in understanding areas for potential development and research. The survey was sent to the Insight Net community and the professional networks of the collabathon planning team. We encouraged all respondents to forward the survey to others. Section 2.2 lists the questions in the survey.

2.1 Survey Results

Twenty-eight individuals completed at least part of the survey. Many did not respond to all of the questions and we report those who responses that we did receive here. Our goal was to obtain insights and qualitative information from individuals beyond our planning team, we just report those responses to questions and do not reporting on missing data. Among the respondents, 16 reported working in higher education and 2 in Public Health. Most respondents had direct experience using R_t tools with 17 reported using R_t estimation tools and 4 had not used these tools. EpiEstim and EpiNow2 were the most commonly used tools with 9 respondents reporting use of them. Among the other packages, EpiFilter was used by 4 respondents, earlyR by 3, epidemia and EpiNowCast by 2 users and the remaining packages by 1 individual.

When asked what they liked about the tools they have used, the following responses were given:

- Parallelization
- EpiEstim - ease of use. EpiNow2 - statistical rigour
- Ease of adoption, R packages, clear documentation and methods

- EpiEstim was great, I also enjoyed the ability to calculate subpopulation Rts and subpopulation to subpopulation exposure rates via ABM data.
- Reproducing the data generating process of reported cases/other data sources
- Easy to use, well documented
- Transparency and information to assist with evaluation
- Ease of use with small and limited data
- I like that I can customize the serial interval and prior values in the EpiEstim package. I also like the forecasting results that EpiNow2 can provide.

When asked about the limitations of the tools, the following responses were given:

- Approach to summarization, ability to change model
- Different input formats
- EpiEstim is dependent on accurate knowledge of the serial interval. ABM data is dependent on computationally expensive models with long development times.
- Not reproducing the data generating process of reported cases/other data sources
- Often limited data on things like serial intervals/etc, also some of the use cases we have (e.g. Rt from wastewater data) involve more complicated measurement models
- Coarse and irregularly reported data. Many tools are not clear about the limitations (e.g., uncertainty propagation of other epi parameters). Some have much tighter confidence intervals than are justifiable. Some ability to capture spatial and hierarchical structure of data along with other variable reporting delays.
- EpiEstim's estimations were unreliable when I used them to assess the Rt for rural counties with low and sporadic case counts. EpiFilter is not a complete package that you have to figure out how to use the functions. EpiNow2 consumes too much computation power. It took too long to run.

The most common challenges individuals mentioned were a lack of worked examples and documentation and slow computational speed. Individuals also mentioned challenges with installing packages. Respondents also mentioned that vignettes and many of the methods were not designed for real-time estimation, but more focused on retrospective estimation.

Figure 2 summarizes the areas that were of greatest interest in the development of R_t. Among those that were deemed most important by respondents were two responses that point to making tools easier to use, including a web interface and software that is easier for non-researchers to use. Other high priority areas included the ability to request new features from developers and implementation in Python. There was also an interest in the development of a community of users.

2.2 Survey Questions

1. Where do you work?
   - University/Higher Education
   - Public Health Department or Agency (e.g. CDC, state or local health department, WHO)

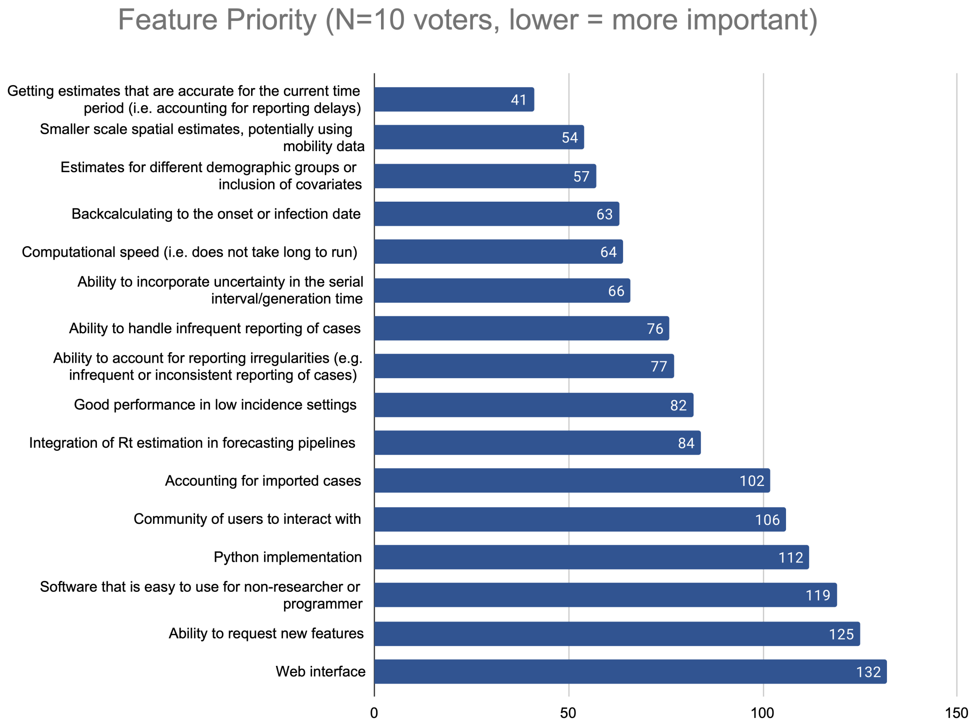

Fig 2. Rankings of areas most important for development of R_t

- Other Government Agency (e.g. NIH)
- Other research institution
- Other, please specify

2. Please provide the name of the institution where you are most likely to use Rt estimation tools.
3. Do you consider yourself:
   - A developer of R_t estimation tools
   - A user of R_t estimation tools
   - Both a user and developer
   - Neither user or developer
4. Have you used R_t estimation tools in the past?
   - No
   - Yes
5. Why have you used R_t estimation tools? (e.g. what pathogen(s), what purpose)
6. What tool(s) have you used (check all that apply)?
   - EpiEstim R package
   - EpiEstim excel spreadsheet
   - EpiFilter
   - EpiNow
   - EpiNow2
   - EpiLPS
   - APEstim
   - bayEStim
   - earlyR
   - Epidemia
   - Other, please specify
7. What features did you like about the tools you have used?
8. What were the limitations of the tool(s) you used?
9. If applicable, what were limitations of tools you did not use that kept you from using that tool?
10. What features would you like to see in future tools to estimate reproductive numbers?
11. What are the challenges you have faced using Rt estimation tools? check all that apply
    - Hard to install
    - Not enough examples or instructions

- Slow computational speed
- Does not work in programming language or software I am most comfortable with
- Does not have features that I need (you can describe here)
- Other (Please specify)

12. What would you prioritize most for future development of R_t estimation tools? (place most important items highest in the list)
    - Computational speed (i.e. does not take long to run)
    - Smaller scale spatial estimates, potentially using mobility data
    - Estimates for different demographic groups or inclusion of covariates
    - Backcalculating to the onset or infection date
    - Getting estimates that are accurate for the current time period (i.e. accounting for reporting delays)
    - Software that is easy to use for non-researcher or programmer
    - Web interface
    - Python implementation
    - Ability to incorporate uncertainty in the serial interval/generation time
    - Ability to handle infrequent reporting of cases
    - Accounting for imported cases
    - Ability to account for reporting irregularities (e.g. infrequent or inconsistent reporting of cases)
    - Good performance in low incidence settings
    - Integration of R_t estimation in forecasting pipelines
    - Community of users to interact with
    - Ability to request new features
